# New approach methods to assess developmental and adult neurotoxicity for regulatory use: a PARC work package 5 project

**DOI:** 10.3389/ftox.2024.1359507

**Published:** 2024-04-26

**Authors:** Tamara Tal, Oddvar Myhre, Ellen Fritsche, Joëlle Rüegg, Kai Craenen, Kiara Aiello-Holden, Caroline Agrillo, Patrick J. Babin, Beate I. Escher, Hubert Dirven, Kati Hellsten, Kristine Dolva, Ellen Hessel, Harm J. Heusinkveld, Yavor Hadzhiev, Selma Hurem, Karolina Jagiello, Beata Judzinska, Nils Klüver, Anja Knoll-Gellida, Britta A. Kühne, Marcel Leist, Malene Lislien, Jan L. Lyche, Ferenc Müller, John K. Colbourne, Winfried Neuhaus, Giorgia Pallocca, Bettina Seeger, Ilka Scharkin, Stefan Scholz, Ola Spjuth, Monica Torres-Ruiz, Kristina Bartmann

**Affiliations:** ^1^ Helmholtz Centre for Environmental Research – UFZ, Chemicals in the Environment Research Section, Leipzig, Germany; ^2^ University of Leipzig, Medical Faculty, Leipzig, Germany; ^3^ Norwegian Institute of Public Health – NIPH, Department of Chemical Toxicology, Oslo, Norway; ^4^ IUF – Leibniz Research Institute for Environmental Medicine, Düsseldorf, Germany; ^5^ DNTOX GmbH, Düsseldorf, Germany; ^6^ Swiss Centre for Applied Human Toxicology, University of Basel, Basel, Switzerland; ^7^ Uppsala University, Department of Organismal Biology, Uppsala, Sweden; ^8^ European Chemicals Agency (ECHA), Helsinki, Finland; ^9^ German Federal Institute for Risk Assessment (BfR), Berlin, Germany; ^10^ Université de Bordeaux, Institut National de la Santé et de la Recherche Médicale (INSERM), Maladies Rares: Génétique et Métabolisme (MRGM), Pessac, France; ^11^ University of Oslo, Section for Pharmacology and Pharmaceutical Biosciences, Department of Pharmacy, Olso, Norway; ^12^ Dutch Nation Institute for Public Health and the Environment (RIVM), Centre for Health Protection, Bilthoven, Netherlands; ^13^ University of Birmingham, Centre for Environmental Research and Justice, Birmingham, UK; ^14^ Norwegian University of Life Sciences (NMBU), Faculty of Veterinary Medicine, Ås, Norway; ^15^ University of Gdansk, Laboratory of Environmental Chemoinformatics, Gdansk, Poland; ^16^ University of Veterinary Medicine Hannover, Foundation, Institute for Food Quality and Food Safety, Hannover, Germany; ^17^ University of Konstanz, In Vitro Toxicology and Biomedicine/CAAT-Europe, Konstanz, Germany; ^18^ AIT Austrian Institute of Technology GmbH, Competence Unit Molecular Diagnostics, Center Health and Bioresources, Vienna, Austria; ^19^ Danube Private University, Faculty of Dentistry and Medicine, Department of Medicine, Krems, Austria; ^20^ Uppsala University and Science for Life Laboratory, Department of Pharmaceutical Biosciences, Uppsala, Sweden; ^21^ Instituto de Salud Carlos III (ISCIII), Centro Nacional de Sanidad Ambiental (CNSA), Environmental Toxicology Unit, Majadahonda, Spain

**Keywords:** new approach method (NAM), developmental neurotoxicity (DNT), adult neurotoxicity (ANT), DNT-IVB, zebrafish, applicability domain

## Abstract

In the European regulatory context, rodent *in vivo* studies are the predominant source of neurotoxicity information. Although they form a cornerstone of neurotoxicological assessments, they are costly and the topic of ethical debate. While the public expects chemicals and products to be safe for the developing and mature nervous systems, considerable numbers of chemicals in commerce have not, or only to a limited extent, been assessed for their potential to cause neurotoxicity. As such, there is a societal push toward the replacement of animal models with *in vitro* or alternative methods. New approach methods (NAMs) can contribute to the regulatory knowledge base, increase chemical safety, and modernize chemical hazard and risk assessment. Provided they reach an acceptable level of regulatory relevance and reliability, NAMs may be considered as replacements for specific *in vivo* studies. The European Partnership for the Assessment of Risks from Chemicals (PARC) addresses challenges to the development and implementation of NAMs in chemical risk assessment. In collaboration with regulatory agencies, Project 5.2.1e (Neurotoxicity) aims to develop and evaluate NAMs for developmental neurotoxicity (DNT) and adult neurotoxicity (ANT) and to understand the applicability domain of specific NAMs for the detection of endocrine disruption and epigenetic perturbation. To speed up assay time and reduce costs, we identify early indicators of later-onset effects. Ultimately, we will assemble second-generation developmental neurotoxicity and first-generation adult neurotoxicity test batteries, both of which aim to provide regulatory hazard and risk assessors and industry stakeholders with robust, speedy, lower-cost, and informative next-generation hazard and risk assessment tools.

## 1 The European Partnership for the Assessment of Risks from chemicals (PARC)

The European Partnership for the Assessment of Risks from Chemicals (PARC) aims to develop next-generation chemical hazard and risk assessment tools to better protect human health and the environment (Marx-Stoelting et al., 2023). A major ambition of the project is to develop new approach methods (NAMs) for human health hazard assessment that covers developmental neurotoxicity (DNT), adult neurotoxicity (ANT), thyroid hormone disruption, immunotoxicity, and non-genotoxic carcinogens. Work package 5.2.1e aims to refine existing NAMs, develop new ones, and generate first-generation ANT and second-generation DNT test batteries. The NAMs that will be developed will be based on Key Events (KE) as identified in the Adverse Outcome Pathway (AOP) framework (Ankley et al., 2010; Leist et al., 2017; Spînu et al., 2021). The work is carried out by a consortium of over 25 experts from 10 EU research institutions and two partner institutions in non-EU countries.

## 2 Exposure to chemicals may pose a risk to the developing and mature nervous systems

Exposure to chemicals can adversely impact nervous system development and function across all stages of life (Costa et al., 2008; Giordano and Costa, 2012; P; Grandjean and Landrigan, 2006). Adverse chemical-dependent effects stemming from exposure of the developing offspring (including *in utero* and postnatal) to the time of sexual maturation may affect the developing nervous system (Costa et al., 2004). Such “developmental neurotoxicity” (DNT) can be long-lasting, extending far beyond the exposure period, and can vary across lifespan (Eriksson et al., 1998; Spalding et al., 2013). Note that any type of neurotoxic effect during development is of regulatory concern and relevant for developmental hazard identification. In contrast, when the mature nervous system is exposed to neurotoxic chemicals, adult neurotoxicity (ANT) effects can be immediate or they may be gradually developing and long-lasting. Depending on the type of ANT effect, it may also be reversible (Spencer and Lein, 2014). Significant and/or severe neurotoxicity, being reversible or irreversible, immediate or delayed, is of regulatory concern.

Due to the sensitivity of the developing nervous system, exposure to low concentrations of certain chemicals may lead to structural and functional disruptions (Rice and Barone, 2000; Grandjean and Landrigan, 2014; Bennett et al., 2016). Neurodevelopmental disorders including autism spectrum disorder, intellectual disability, attention deficit/hyperactivity disorder (ADHD), neurodevelopmental motor disorders (including tic disorders), and specific learning disorders can have lifelong socioeconomic consequences, including diminished economic productivity or an increased need for learning support in schools (P. Grandjean and Landrigan, 2006). Whilst estimates were acknowledged to be uncertain, in the EU, ∼30,000 disability adjusted life years (DALYs) related to neurodevelopmental disease may be the result of chemical exposure (and irrespective of a person’s genetic predisposition/sensitivity), with ∼250,000 DALYs when chemical exposure was combined with underlying genetic predisposition (EC 2019). This estimate was based on a ‘top down’ assessment of impacts of pervasive neurodevelopmental disorders from the World Health Organization (WHO) and an estimate that 3% is due to environmental exposure to legacy compounds such as lead and other environmental pollutants (EC 2019). Notable socioeconomical benefits are therefore predicted via the identification of substances which are known or presumed to cause DNT and subsequent prevention of exposure (Bellanger et al., 2013; Bellanger et al., 2015).

After the developmental period, acute and/or chronic exposure to environmental chemicals may elicit toxic responses in the peripheral and/or central nervous systems and it has been suggested that exposure to specific chemical agents may increase the probability of developing neurodegenerative disorders such as Parkinson’s and Alzheimer’s Diseases, or dementia (Landrigan et al., 2005; Tanner et al., 2014; Ockleford et al.). Moreover, exposure to certain chemicals has been suspected to be linked to adolescent and adult depression, anxiety, and other psychiatric disorders in a number of academic publications (Dickerson et al., 2020; Hollander et al., 2020; Jacobson et al., 2022; Rokoff et al., 2022; Aung et al., 2023). In a study of 22 chemical inventories from 19 countries and regions, over 350,000 chemicals and mixtures of chemicals were identified as registered for production and potentially in use (Wang et al., 2020). Despite knowledge concerning the potentially harmful impacts of environmental chemicals on the developing and mature nervous systems (P. Grandjean and Landrigan, 2006), it is understood that only a limited number of unique substances has been tested for DNT using OECD Test Guideline (TG) studies. (OECD, 2008a; Makris et al., 2009; Sachana et al., 2019; Crofton and Mundy, 2021).

## 3 Policy and regulatory landscapes

The EU Green Deal describes health impacts in the Zero-Pollution Action Plan, and the European Commission recently highlighted their interest in increased efforts to protect against the most harmful chemicals, by further exploring the risk management possibilities of neurotoxic and endocrine disrupting (which has been linked to DNT) substances (European Commission, 2020). In the EU, several relevant regulations are in force. For example, before entering the market or gaining approval as a biocidal or pesticidal active substance, the minimum data requirements described in the relevant EU Regulation must be fulfilled (among other conditions). EU regulations on plant protection products (Reg EC 1107; European Parliament and Council, 2009) and biocides (Reg EC 528; European Parliament and Council, 2012) can require DNT/ANT testing as part of the data requirements. Under the EU Biocides Product Regulation (Reg EC 528; European Parliament and Council, 2012), specific DNT testing, for example, OECD TG 426, recently became a mandatory information requirement for the approval process of active biocidal substances. Under REACH (European Parliament and Council, 2006), the European Regulation created to protect human health and the environment from harmful chemicals, the level of information required to identify potential neurotoxic (DNT/ANT) properties currently depends on the tonnage and identification of specific concerns that may trigger DNT or ANT tests. The available information is used to apply appropriate hazard classifications, as per the criteria specified in the CLP regulation (Reg EC 1272; European Parliament and Council, 2008), to inform on the hazardous properties of chemicals. Classification in accordance with CLP then serves to trigger or inform remedial actions in other legislation to control the hazard. The CLP regulation (Articles seven and 8) does not require DNT or ANT TGs directly but rather makes use of all available data generated in the context of relevant legislation and/or otherwise available in the public domain. In cases where such data is not available to inform on a given hazard, testing may be conducted under certain conditions including the condition that tests on animals are to be carried out only where no other alternatives, which provide adequate reliability and quality of data, are possible. This implies support and presents an opportunity for the development, validation, and implementation of NAMs.

Within the CLP Regulation, substances with DNT are addressed under the reproductive (developmental) toxicity hazard class and ANT effects are addressed under Specific Target Organ Toxicity (STOT), either single exposure (SE) or repeated exposure (RE), depending on whether the effects are caused by single or repeated exposures, respectively. The recent revision of the CLP regulation includes a new hazard class for endocrine disruption that includes endocrine activity mediated adverse effects on the developing (and mature) nervous system (Reg EC 1272; (European Parliament and Council, 2023). According to the new criteria, classification as ED Category one shall be largely based on evidence from at least one of the following: human data; animal data; non-animal data providing an equivalent predictive capacity as human data or animal data (Reg EC 1272; European Parliament and Council, 2023). Thus, the new hazard class allows for NAMs to be directly used for the purpose of this specific classification when the criteria are met.

### 3.1 DNT/ANT in current chemical regulations

The information needed to fulfill the data requirements under REACH and BPR is typically provided by the *in vivo* OECD TG studies defined in the relevant section of the applicable regulation, but there are also specific possibilities for adaptation (more specifically data waiving). Such adaptation possibilities can include non-animal approaches and/or the use of existing information stemming from similar substances via a read-across approach (European Parliament and Council, 2006). However, where data on human health and environmental properties are derived via adaptations to data requirements, certain conditions apply. The conditions for adaptations using *in vitro* data under REACH are specified in Annex XI, section 1.4. In the context of a read across adaptation (REACH Annex XI, section 1.5), again certain restrictive conditions apply with regard to the data that directly informs on the hazard. However, for the extrapolation of such data to other substances, there is a clear opportunity to utilize NAMs as supportive information to demonstrate similarity in the properties of the substances concerned.

It should also to be noted that, depending on the applicable regulation, new tests may not be necessary if the available data is already sufficient for the regulatory purpose as given in the specific regulation. For example, the DNT study shall not be conducted under the BPR if the available data already indicate that the substance causes developmental toxicity and meets the criteria to be classified as toxic for reproduction category 1A or 1B: May damage the unborn child (H360D), and these available data are adequate to support a robust risk assessment (European Parliament and Council, 2012).

A range of OECD TG studies, including single-dose studies (e.g., OECD TG 402, 403, 420, 423, 425) and/or repeated dose toxicity studies (e.g., OECD TG 407, 408, 421, 422, 414, 443 in the absence of DNT cohorts) may inform on ANT or DNT based on clinical signs such as paralysis, convulsions, lack of coordination, or ataxia or neurohistopathology and/or alterations in brain weight (Table 1). DNT can be evaluated more comprehensively using dedicated tests such as OECD TG 426 or in the DNT cohort (cohorts 2A and 2B) of the Extended One-Generation Reproduction Toxicity Study (EOGRTS, OECD TG 443). OECD TGs dedicated to studying ANT include OECD TG 424, 418 and 419. Under REACH, ANT or specific mechanisms/modes of action with an association to (developmental) neurotoxicity can be used to trigger specific DNT studies. Substances in food intended for infants can also prompt investigations to assess potential DNT (EFSA Scientific Committee et al., 2017).

**TABLE 1:** Description of existing OECD guideline studies that include neurotoxicity as an endpoint.

Test guideline	Primary endpoint	Neurotox endpoint	Preferred Species	Administration period	Non-behavioral endpoints related to neurotoxicity	Behavioral endpoints	Reference
OECD TG 402	Dermal Toxicity	ANT (acute)	Rat	Adults (<24 h)	No (just gross necropsy)	Autonomic and central nervous system and somatomotor activity and behavior pattern	OECD (2017)
OECD TG 403	Inhalation Toxicity	ANT (acute)	Rat	Adults (4 h)	No (just gross necropsy)	Autonomic and central nervous system and somatomotor activity and behavior pattern	OECD (2009)
OECD TG 407	Oral Toxicity/Endocrine Disruption	ANT (chronic)	Rat	Adults (daily - 28d)	Brain weight, histopathology of brain, spinal cord, and sciatic nerve	Sensory reactivity to stimuli, limb grip strength, motor activity	OECD (2008b)
OECD TG 408	Oral Toxicity/Endocrine Disruption	ANT (chronic)	Rat	Adults (daily - 90d)	Brain weight, histopathology of brain, spinal cord, and sciatic nerve	Sensory reactivity to stimuli, limb grip strength, motor activity, autonomic activity	OECD (2018a)
OECD TG 418	Neurotoxicity (OP substances)	ANT (acute)	Hen	Adults (single dose)	Neuropathology of central and peripheral nervous system, NTE and AchE activities	Behavioral abnormalities, ataxia, and paralysis	OECD (1995a)
OECD TG 419	Neurotoxicity (OP substances)	ANT (chronic)	Hen	Adults (≥28 days)	Neuropathology of central and peripheral nervous system, NTE and AchE activities	Behavioral abnormalities, ataxia, and paralysis	OECD (1995b)
OECD TG 420	Oral Toxicity	ANT (acute)	Rat	Adults (single dose)	No (just gross necropsy)	Somatomotor activity and behavior patterns	OECD (2002a)
OECD TG 423	Oral Toxicity	ANT (acute)	Rat	Adults (single dose)	No (just gross necropsy)	Somatomotor activity and behavior patterns	OECD (2002b)
OECD TG 424	Neurotoxicity	ANT (chronic)	Rat	Adults (≥28 days)	Neuropathology of central and peripheral nervous system	Sensory reactivity to stimuli, limb grip strength, motor activity	OECD (1997)
OECD TG 425	Oral Toxicity	ANT (acute)	Rat	Adults (single dose)	No (just gross necropsy)	Somatomotor activity and behavior patterns	OECD (2022)
OECD TG 426	Neurotoxicity	DNT (chronic)	Rat	Gestation & Lactation	Developmental abnormalities, Brain weights, Neuropathology	Motor activity, Motor and sensory function, Learning and memory	OECD (2007)
OECD TG 443	Reproductive Toxicity	DNT (chronic)	Rat	Premating - Pups	Neurohistopathology, Brain weight and morphometry	Auditory startle, Functional observational battery (open field, manipulative, and physiologic), motor activity	OECD (2018b)

NTE: neuropathy target esterase; AchE: acetylcholinesterase; M: male; F: female.

As the development of the nervous system starts prenatally and continues to develop through adolescence, reaching adult levels of neurotransmitters, synaptic plasticity, myelination and grey matter at around age of 20 in humans and around PND60 in rats (Semple et al., 2013), it is key to implement exposure throughout the whole developmental period for improving the chances to identify developmental neurotoxicants. In an OECD TG 426, the offspring are exposed as a minimum from the time of implantation (starting on gestation day (GD) 6) throughout lactation (until postnatal day (PND) 21). In cohort 2A of an EOGRTS, the offspring are exposed via the mother *in utero*, through lactation and directly at least after weaning until termination on ∼ PND 66–77. The assessed DNT parameters in specific DNT studies include as a minimum (depending on the OECD TG) motor activity, motor and sensory function, associative learning and memory (only in OECD TG 426 as standard testing), brain weight, and central and peripheral nervous system histopathology (Tsuji and Crofton, 2012). As dedicated DNT studies are often complex studies using rodents, they are resource-intensive regarding time, costs, and number of animals (Crofton et al., 2012; Smirnova et al., 2014) and only a limited number of chemicals have been tested for DNT using OECD TG DNT studies (OECD, 2008a; Makris et al., 2009; Sachana et al., 2019; Crofton and Mundy, 2021). In addition, variability in rodent neurotoxicity tests has been documented (Tsuji and Crofton, 2012; Terron and Bennekou, 2018; Sachana et al., 2019; Paparella et al., 2020; Harry et al., 2022) which indicates a need to specify, optimize, and harmonize the individual test methods used as part of the OECD TG. It also underscores the need for new tests that lack the excess variability associated with *in vivo* guideline studies for the assessment of DNT.

The risk posed by unidentified (developmental) neurotoxic agents and the limited number of timely and cost-efficient test systems (i.e., NAMs) serve as the basis for this PARC project where the ambition is the generation of an improved *in vitro* and alternative DNT test battery and a first-generation *in vitro* ANT test battery. The need to develop NAM-based next-generation hazard and risk assessment for DNT and ANT has found international support from academic scientists, industry, certain regulatory authorities, and other interest groups (Smirnova et al., 2014; Ockleford et al., 2017; Fritsche et al., 2018; Kavlock et al., 2018; Craig et al., 2019; Paparella et al., 2020; Vinken et al., 2021; Pallocca et al., 2022; Stucki et al., 2022). ECHA recently published *Key Areas of Regulatory Challenge* (ECHA, 2023), which highlights several of the known scientific and regulatory challenges that NAMs face. It further underlines the need for additional research in the field of ANT and DNT NAMs (ECHA, 2023).

NAMs have been advocated to be implemented into the regulatory hazard assessment stage of chemicals risk assessment (Stucki et al., 2022; Schmeisser et al., 2023). Currently, *in vitro* data may be used in Weight of Evidence assessment for classification and labelling (e.g., for developmental toxicity), or to trigger further DNT tests at REACH Annex IX and X, or to support grouping and read across from similar substances. High-throughput *in vitro* assays have also great potential as screening tools to prioritize chemicals and specific modes of action (MoA) for further testing (Escher et al., 2023). While such high throughput screening (HTS) tools have not yet been implemented for DNT and ANT in large scale regulatory practice, the introduction of more sophisticated *in vitro* tests and the validation of all HTS assays for DNT and ANT appear vital to improve their regulatory usefulness. It has been suggested that new *in vitro* methods should be mechanistically associated with adverse (developmental) neurotoxic outcomes (Pitzer, Shafer, and Herr, 2023). This is important to establish the toxicological relevance of endpoints measured in NAMs and/or to allow for the selection of the most informative follow-up studies to produce new information to elicit regulatory action (Smirnova et al., 2014; Ockleford et al., 2017; Fritsche et al., 2018; Kavlock et al., 2018; Craig et al., 2019; Paparella et al., 2020; Vinken et al., 2021; Pallocca et al., 2022; Stucki et al., 2022).

## 4 Building the DNT-IVB v2.0

**FIGURE 1:**
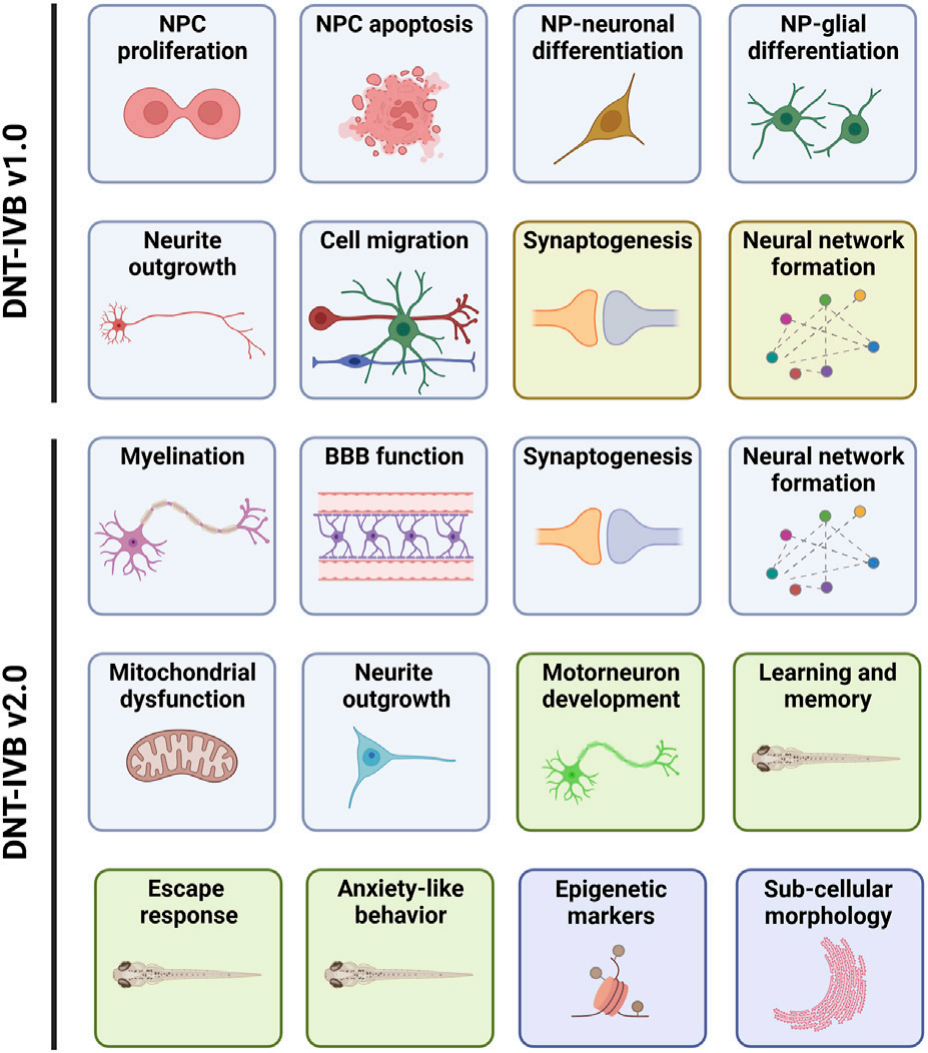
The DNT-IVB. Version 1.0 of the DNT-IVB (Masjosthusmann et al., 2020; Blum et al., 2022; OECD, 2023) contains endpoints for human neural progenitor cell (hNPC) proliferation and apoptosis, cell (neuronal, radial glia, oligodendrocytes) migration, hNPC-neuronal and oligodendrocyte differentiation, neurite outgrowth, neuronal maturation and synaptogenesis, and neuronal network formation (top). While most assays are conducted in human cells (blue boxes), two are performed in rat primary cells (yellow boxes). PARC 5.2.1e builds and evaluates NAMs for key battery gaps including myelination, blood-brain barrier (BBB) formation, mitochondrial function, and a suite of automated behavior-based NAMs in early life-stage zebrafish (green boxes). In addition, replacement of rodent-based NAMs with human-based test systems is also underway. Lastly, early epigenetic or sub-cellular morphological indicators of later DNT-related effects are being generated and evaluated for potential use in the DNT-IVB v2.0. Adapted from Crofton and Mundy, 2021.

One of the purposes of this PARC project is to deliver a guidance document containing a framework to facilitate the regulatory use of data derived from a DNT *in vitro* NAM-based test battery. A basic DNT *in vitro* test battery (IVB) has already been developed (i.e., DNT-IVB v1.0). It covers several cellular neurodevelopmental processes vital for normal brain development (Bal-Price et al., 2018; Masjosthusmann et al., 2020; Crofton and Mundy, 2021; Blum et al., 2022; Koch et al., 2022; OECD, 2023). The DNT-IVB v1.0 (Figure 1) measures effects of chemicals on human neural progenitor cell (hNPC) proliferation (Baumann et al., 2014; Baumann et al., 2015; Harrill et al., 2018; Nimtz et al., 2019; Masjosthusmann et al., 2020; Koch et al., 2022) and apoptosis (Druwe et al., 2015; Harrill et al., 2018), cell migration (Baumann et al., 2015; Baumann et al., 2016; Nyffeler et al., 2017; Schmuck et al., 2017; Masjosthusmann et al., 2020; Koch et al., 2022), hNPC-neuronal differentiation (Baumann et al., 2015; Schmuck et al., 2017; Masjosthusmann et al., 2020; Koch et al., 2022), oligodendrocyte differentiation (Dach et al., 2017; Schmuck et al., 2017; Masjosthusmann et al., 2020; Klose et al., 2021; Koch et al., 2022), neurite outgrowth (human: Harrill et al., 2010; Harrill et al., 2018; Krug et al., 2013; Hoelting et al., 2016; Masjosthusmann et al., 2020; Koch et al., 2022; rat; Harrill et al., 2013; Harrill et al., 2018), and synaptogenesis and neuronal network formation (rat: Harrill et al., 2011; Harrill et al., 2018; Brown et al., 2016; Frank et al., 2017; Shafer, 2019).

Gap analysis of the DNT-IVB v1.0 revealed a requisite for supplementary cell assays (e.g., microglia) and functions (e.g., human neuronal network formation, astrocyte function, behavior, learning, and memory) to augment coverage and increase the ability to detect potential (developmental) neurotoxicants (Crofton and Mundy, 2021). Coverage of additional targets for neurotoxicants (e.g., signaling pathways and processes) is necessary, as exemplified by nicotine, a compound identified as a false negative in the DNT-IVB v1.0 (Masjosthusmann et al., 2020; Crofton and Mundy, 2021; Blum et al., 2022). This indicates the inability to detect (developmental) neurotoxic compounds that target nicotinic receptors in these test systems (e.g., neonicotinoid insecticides) (Sheets et al., 2016; Loser et al., 2021; Blum et al., 2022). To address some of the identified gaps, there are four key areas that this PARC project aims to improve during the development of the DNT-IVB v2.0 (Figure 1). This includes refinement of existing assays, generation of new NAMs to cover essential gaps, determination of the applicability domain for relevant available NAMs, and increased cost efficiency.

### 4.1 Refine existing assays

The current synaptogenesis and neural network formation assays were based in primary rat cortical cells differentiated in 2D on multielectrode arrays (MEA; Brown et al., 2016; Frank et al., 2017) (Figure 1). While there is also a recently established human neural network formation (hNNF) assay (Bartmann et al., 2023), it requires commercially available human-induced pluripotent stem cells (hiPSCs), which are used to derive excitatory and inhibitory neurons and primary human astrocytes that can be plated on MEAs for a functional assessment of network formation. This assay has recently been used to evaluate the effects of pesticides on human neural network formation (Bartmann et al., 2023). To decrease the costs of the hNNF assay, the PARC consortium will re-establish and refine the protocol using non-commercially generated hiPSC-derived excitatory and inhibitory neurons, together with human astrocytes derived from hNPCs (Koch et al., 2022). Synapse assembly is a critical feature of neurodevelopment. The DNT-IVB v1.0 assay for synaptogenesis is currently based on primary rat cortical cells (Harrill et al., 2011; Harrill et al., 2018). Human iPSC-derived NPCs can be differentiated into different types of postmitotic neurons and astrocytes (Davidsen et al., 2021; Lauvås et al., 2022). Therefore, a test system, comprised of a 2D mixed culture of neurons and astrocytes undergoing differentiation, will be developed and refined using high-content imaging. To enable comparison to data generated in the rat synaptogenesis assay, a chemical test set will be evaluated (described below).

Another identified gap within the DNT-IVB v1.0 is a lack of assays that describe mitochondrial toxicity events in susceptible cell types. AOP3 (“inhibition of the mitochondrial complex I of nigro-striatal neurons leads to parkinsonian motor deficits”) describes a link between inhibition of complex I of the mitochondrial respiratory chain and motor deficits associated with parkinsonian disorders (https://aopwiki.org/aops/3). The current DNT-IVB v1.0 assays are not particularly sensitive or fail to detect known mitochondrial toxicants (Masjosthusmann et al., 2020; Crofton and Mundy, 2021). To increase the sensitivity of battery assays to this class of neurotoxicants, several DNT-IVB v1.0 assays will be modified to allow for increased detection of mitochondrial toxicants. This step includes the assessment of neurite area and cell viability in human dopaminergic neurons and human immature peripheral neurons. While these endpoints are covered in the DNT-IVB v1.0, where the NAMs are performed in glucose-containing medium, in DNT-IVB v2.0, the assay will be performed in glucose-free, galactose-containing medium, which makes cells more reliant on their mitochondria and increases their sensitivity to mitochondrial toxicants (Hoelting et al., 2016; Delp et al., 2019; Delp et al., 2021).

### 4.2 Build new NAMs to cover essential gaps

#### 4.2.1 Cellular gaps

In a key analysis, 29 neurotoxicity MoAs were characterized for 248 individual compounds representing 23 compound classes and 212 natural neurotoxins (Masjosthusmann et al., 2018). More comprehensive assessment of the potential for chemicals to harm the developing nervous system likely requires NAMs that cover the identified MoAs. One MoA not covered in the DNT-IVB v1.0 is the formation of a functional blood-brain barrier (BBB). The BBB determines the ability of some environmental chemicals to reach the central nervous system (Banks, 2009). Chemical exposure can affect the BBB integrity to cause DNT effects (Saili et al., 2017). Here, we will develop and use an hiPSC-based BBB NAM to test whether chemical exposure increases permeation of chemicals across the barrier, resulting in higher concentration reaching the central nervous system. According to established differentiation protocols (Appelt-Menzel et al., 2017), chemicals will be applied during cellular differentiation and transendothelial electrical resistance will be used as a readout of barrier function. One MoA considered relevant for DNT and not yet covered by the DNT-IVB v1.0 is the contribution of inflammatory reactions of glial cells. The key cell populations producing inflammatory mediators in the brain are astrocytes and microglia (Carson et al., 2006). These cells can be generated from human stem cells (Brüll et al., 2020; Spreng et al., 2022) and then either tested as pure populations, as mixed glial populations or together with various neuronal cultures (Gutbier et al., 2018; Klima et al., 2021).

#### 4.2.2 Functional gaps

OECD TG 426 (OECD, 2007) assesses neurobehavioral endpoints which include measures of cognition (including associative learning and memory) in rodents exposed to chemicals during the developmental period. Cellular NAMs may provide information on cellular events that may eventually cause adverse effects on cognitive functions or other neurobehavioral functions but fail to provide equivalent information to neurobehavioral tests. In addition, when considering the complex integration of intracellular, intercellular, interregional, and systemic interactions that occur in development-stage and regional specific manners, *in vitro* NAMs do not cover all relevant cell types and processes, inherent within whole organisms, that are necessary to develop and maintain a functional nervous system. In this project, the alternative (i.e., relative to rodent models) early life zebrafish model will be used to generate a range of behavior-based assays that complement the *in vitro* approaches described above.

Zebrafish (*Danio rerio*) are a 3Rs-compliant (Hooijmans et al., 2010), non-protected vertebrate model up to 5 days post fertilization (dpf) (Strähle et al., 2012; Kalueff et al., 2013). The zebrafish embryo model may represent a powerful translational system for human hazard and risk assessment as zebrafish possess orthologs for 70% of human genes (Howe et al., 2013), 80% of human disease-related genes (Howe et al., 2013), and 86% of general human drug targets (Gunnarsson et al., 2008). Zebrafish are increasingly being utilized as a model system to investigate the function of the growing list of risk genes associated with neurodevelopment disorders (Sakai et al., 2018), including motor neuron diseases (Babin et al., 2014). Zebrafish neurodevelopment starts at 24 h post-fertilization and primary neurogenesis is complete by roughly 72 h post-fertilization (depending on rearing temperature). Resulting neuroanatomy (Gupta et al., 2018), nervous system transcriptomic lineages (Raj et al., 2018), and brain asymmetry (Duboc et al., 2015) are suggested to be comparable to humans. In addition, neurotransmitter systems, including glutaminergic, cholinergic, serotonergic, dopaminergic, adrenergic, GABAergic, and histaminergic (Babin et al., 2014; Faria et al., 2015; Horzmann and Freeman, 2016) are similar to those found in humans and associated with sensory-motor outcomes. This rapid establishment of neural structures during neurodevelopment and their link to quantifiable behavioral parameters is a major asset for PARC WP5.2.1e.

Relative to *in vitro* systems, metabolically competent zebrafish embryos may address potential toxicokinetics that can affect toxicity outcomes (Chu and Sadler, 2009). Regarding neurotoxicity, the assessment of neurobehavioral effects caused by xenobiotic exposure is advantageous because these perturbations are sensitive (i.e., they occur at sub-morphological concentrations) (Noyes et al., 2015; Bruni et al., 2016; Gaballah et al., 2020; Jarema et al., 2022). Locomotor activity can function as an automated and generalized readout of neurodevelopment. A major advantage of the early life zebrafish system as compared to *in vitro* systems is that it represents an alternative whole organism animal system that is emendable to genome-wide differential expression data collection throughout early neurodevelopment (Kettleborough et al., 2013) and are expected to address comparatively more MIEs and KEs related to DNT in a single assay (e.g., neurotransmitter signaling pathways, functional BBB, myelinated axons, functional synapses, neuronal networks, and neural circuits), as compared to individual *in vitro* test systems performed in single cell types or limited co-culture systems. Moreover, zebrafish development has been mapped at the resolution of single-cell transcriptomics, allowing the detection of cell-type specific changes associated with chemical induced adversity affecting neural and non-neural components of the developing brain (Farrell et al., 2018).

In compliance with EU directive (2010/63/EU; European Parliament and Council, 2010), the majority of work will be conducted in embryos up to 5 dpf. Another key advantage of early life stage zebrafish NAMs is that they can be screened in DNT and acute modes by varying the length and timing of chemical exposure. The DNT mode captures structural and functional deficits that alters locomotor activity in response to various stimuli. The acute mode identifies rapid, receptor-mediated changes in neuroactivity that can potentially be used as a complement to cellular ANT assays which aim to identify perturbations in signaling pathways (e.g., dopaminergic signaling) linked to ANT AOPs.

All DNT NAMs performed in early life-stage zebrafish described will be used following developmental exposure to PARC test chemicals and removed prior to behavior testing. This increases the likelihood of detecting functional or structural effects that arise from developmental perturbations in underlying behavior circuits after chemical exposure has ceased. Later, in the development of the ANT-IVB 1.0, the same assays will be applied post-neurogenesis (after three dpf) to detect the acute neuroactivity potential of test chemicals with a focus on the detection of perturbations in receptor-based neurotransmitter systems that are associated with DNT and/or ANT (e.g., dopaminergic, gabaergic, glutamatergic perturbations).

**FIGURE 2:**
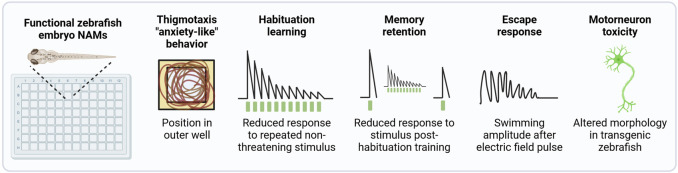
Functional NAMs performed in early life stage zebrafish. A suite of automated zebrafish-embryo behavior-based NAMs are under development for potential inclusion in the DNT-IVB v2.0. All NAMs are performed using automated tracking. Most assays will be performed in 96 square well plates for a comparable throughput to *in vitro* assays. Based on the exposure paradigm used, these assays can be performed with protocols that predict DNT and/or ANT. For the detection of DNT, chemical exposure occurs during development and is removed prior to behavior assessments. In contrast, if exposure takes place after neurogenesis is complete, any adverse effect related to the specific signalling pathways that have reached maturation may be indicative of acute ANT. However, as the organism’s nervous system is still developing, it cannot be excluded that the nature of the effect may be considered within a DNT assessment framework.

Another important functional topic is the impact of chemical exposure on associative learning and memory, which is assessed as a standard part of rodent-based OECD TG 426 for DNT (OECD, 2007), and may be included as an add on to TG 424 (OECD, 1997) for ANT and in TG 443 for DNT (OECD, 2018b). *In vitro* test systems are unable to account for these complex behavioral and cognitive aspects. Members of this consortium are developing a NAM that detects chemical-dependent disruption of non-associative learning and memory retention in early life stage zebrafish (Figure 2). An escape response NAM that identifies chemicals that specifically disrupt the motor system via the activation of reticulospinal neurons and independently of sensory processing (Dubrana et al., 2021; Knoll-Gellida et al., 2021) will be further developed and applied to screen a common set of chemicals described below. A NAM for chemical-induced developmental motor neuron toxicity will also be developed using the transgenic line Tg (nrp1a:gfp)js12 with labeled motor neurons (Sato-Maeda et al., 2006). A NAM that detects anxiety-like behavior via the detection of thigmotaxis, or the time spent along the outer edge of a well, is also under development. Finally, for a subset of test chemicals, the persistence of behavioral effects, post-exposure, will be evaluated to substantiate the detection of DNT effects in 14 dpf old larvae.

#### 4.2.3 Determine applicability domain

The applicability domain describes the physicochemical or other properties of the chemicals for which a NAM is applicable for use (OECD, 2005). The applicability domain is generally determined using a range of reference chemicals linked to an adverse effect (OECD, 2005). Within this project, a test set of 96 reference chemicals, based on previously published work (Masjosthusmann et al., 2020; Blum et al., 2022), will be evaluated. With the exception of the zebrafish NAMs, all other DNT NAMs will be performed in human cellular assays. As these models contain a limited number of cell types and other gaps, efforts will be made to assess the applicability domain to determine whether, and to what extent, the NAMs cover established DNT AOPs, including endocrine disruption (ED) or proposed DNT AOPs such as epigenetic perturbation. Specifically, in the 2D synaptogenesis model, characterization of differentiation up to 28 days will be performed to understand the abundance and distribution of ED-relevant receptors including retinoic acid receptor, estrogen receptor, androgen receptor, thyroid hormone receptor, glucocorticoid receptor and liver X receptor. Coverage of ED and epigenetic modes of action will also be carried out for certain cellular ANT NAMs (see below).

#### 4.2.4 Increase molecular and cellular coverage, reliability, and cost efficiency

Mammalian TG studies for DNT testing are costly and time-consuming (Crofton et al., 2012; Smirnova et al., 2014). At the same time, mammals contain a complete nervous system with all functional components throughout the whole developmental period, and their communication with other relevant organs and systems, (e.g., gut, liver, endocrine, and immune systems) that can collectively influence neurotoxicity outcomes. Next-generation DNT and ANT testing seeks to eventually replace mammalian tests with a battery of *in vitro* and alternative test systems. Some of these next-generation test systems are lengthy and can take up to 35 days, which increases the overall cost of the potential test battery. To complement cellular and alternative assays potentially included in the DNT-IVB v2.0 and to provide a low-cost screening strategy, four approaches will be explored.

The first strategy seeks to identify early markers of later DNT-associated KEs with a focus on epigenomic or sub-cellular morphological alterations. Epigenomic processes drive cell differentiation, and chemically induced alteration in epigenetic patterns can lead to long-term changes in gene function (Baccarelli and Bollati, 2009). In this context, we will assess whether epigenetic re-arrangements precede morphological changes observed in differentiation-related assays, with the potential to shorten and/or strengthen such assays. This step will be addressed by performing and comparing different genome-wide epigenetic (i.e., DNA methylation) analyses at specific time points in cellular differentiation assays where shortening could be of interest. Cell painting is a high-throughput microscopy technique that allows researchers to simultaneously label and visualize multiple organelles in a cell (Bray et al., 2016). It uses a cocktail of fluorescent stains and high-content imaging to obtain parallel morphological measurements on a single cell level. This project will establish and evaluate an automated cell profiling methodology for DNT assays that is cheap, fully automated, data-rich, and can operate on 2D cell monolayers, and work is ongoing to expand to 3D spheroids. Images from Cell Painting are data-rich and highly applicable for analysis with artificial intelligence methods, for example, for MoA prediction (Tian et al., 2023), and assessment of combination effects of environmental compounds (Rietdijk et al., 2022). Morphological changes will be assessed as potential early indicators of DNT or ANT outcomes in short- and longer-term cellular assays.

The second strategy employs transcriptomics to refine the search for early markers of later DNT-associated KEs with a focus on genome-wide expression patterns observed at a single cell resolution of intact developing embryo nervous systems. This single-cell transcriptome data is complemented by building transcriptomics and metabolomics technologies into an existing OECD TG for embryotoxicity (TG 236) to measure (neuro)developmental toxicity endpoints. While it was developed as one of the potential alternatives to the acute test on fish (OECD TG 203) for ecotoxicological hazard assessment, it has been reported that compared to OECD TG 203, OECD TG 236 may underestimate acute toxicity for certain types of chemicals, in particular neurotoxicants (Klüver et al., 2016; Glaberman et al., 2017; Sobanska et al., 2018). Nevertheless, OECD TG 236 has recently attracted considerable attention for its potential expansion to human health endpoints, particularly developmental (neuro) toxicity (Braunbeck et al., 2014; Krzykwa et al., 2019).

The third strategy uses a rapid, low-cost cellular neurite outgrowth assay to screen a much larger chemical test set, including human-relevant mixtures (J. Lee et al., 2022a; J; Lee et al., 2022b). For this assay, SH-SY5Y cells, sub-cloned from a neuroblastoma cell line, are used in 384 well plate format (J. Lee et al., 2022b; J; Lee et al., 2022b). The specificity and sensitivity of this assay will be compared to non-transformed, hiPSC-derived DNT models.

The fourth strategy will build *in silico* models to predict the probability of inducing DNT effects using Quantitative Structure-Activity Relationships (QSAR) models to link chemical structural properties with measured neurotoxicity effects (Khelfaoui et al., 2021; Grillberger et al., 2023). Molecular docking combined with observed molecular dynamics will be employed to reflect interactions of organophosphates with cellular targets (e.g., membranes, proteins) identified as MIEs according to DNT-relevant AOPs (Gadaleta et al., 2022). Serine esterases and calcium transporters are currently under consideration (Knoll-Gellida et al., 2021). Method evaluation will be performed by comparing simulations with experimentally generated data. Binding affinity for the set of targets will then be predicted by using machine learning-based models, and structural alerts for pathway perturbation will be identified.

## 5 Demonstration of added value and identification of a minimum assay battery for DNT-IVB v2.0

A fundamental requirement for the regulatory acceptance of NAMs involves the development of test methods characterized by a high degree of robustness, performance, and readiness (Bal-Price et al., 2018), including acceptable levels of variability (Harry et al., 2022). If they are intended for future use in the context of hazard assessment, NAMs should ideally meet or exceed the sensitivity, specificity, accuracy, and reliability of the respective OECD TG, to ensure a continued level of acceptable chemical safety (Bal-Price et al., 2018). This approach ensures the use of data with a significant level of confidence. In the case of the DNT-IVB v1.0, elevated standards of readiness and robustness have already been demonstrated (Bal-Price et al., 2018; Krebs et al., 2019; Blum et al., 2022). To assess DNT-IVBv1.0 performance, a set of 45 reference (i.e., performance) compounds, consisting of 28 substances that were considered DNT positive and 17 substances that were considered DNT negative by the assay developers were used. Using these substances, an assay sensitivity of 68%, specificity of 100%, and accuracy of 80% was observed (Blum et al., 2022).

To substantiate the added value of new and refined assays within the DNT-IVB v2.0, a 96-member reference set will be used, which encompasses 45 performance compounds from the DNT-IVB v1.0 (Blum et al., 2022) and will be augmented with known modifiers targeting pathways specific to DNT (Fritsche, 2017) (e.g., mTOR (Lee, 2015), PDGFR-PLCγ1 (Kang et al., 2016), Notch (Imayoshi et al., 2013), and thyroid hormones (TH; López-Espíndola et al., 2014; Bernal et al., 2015)). Centralized chemical procurement and distribution will occur via a collaborative effort involving PARC 5.2.1e scientists and experts from the EU Joint Research Centre (EU-JRC). This structured approach facilitates standardized and comparable assessment of substances across partner laboratories, effectively minimizing uncertainties associated with substance purity, solubilization, and concentration. By testing the DNT-IVB v1.0 performance compounds at reasonable concentrations in each newly developed test method, we will gain insight into the chemical applicability domain of each assay to understand the potential added value of DNT-IVB v2.0 NAMs. If DNT-IVB v2.0 NAMs can appropriately identify DNT-IVB v1.0 false negatives as positives, this will increase the sensitivity of the resulting v2.0 test battery. In addition, the evaluation of specific pathway agonists and antagonists will reveal the applicability domain of each assay and the whole DNT-IVB v2.0.

## 6 Build ANT-IVB v1.0

In contrast to DNT, there is no comparable set of NAMs with a high readiness for ANT testing that covers important MoA. ANT can be elicited through a variety of mechanisms involving neurotransmitter receptors and ion transporters which influence the transmission and processing of signals in the human brain and other parts of the nervous system (Fritsche and Hogberg, 2020; Masjosthusmann et al., 2018). Recently, a neurotoxicity MoA analysis was performed for 248 individual compounds, representing 23 compound classes and 212 natural neurotoxins (Masjosthusmann et al., 2018). The identified MoA were grouped according to ANT common key events including cholinergic, GABAergic, glycinergic, glutamatergic, adrenergic, serotonergic, and dopaminergic neurotransmission, ion channels/receptors (e.g., sodium channels, potassium channels, calcium channels, chloride channels), and a range of cellular endpoints such as mitochondrial dysfunction, oxidative stress, apoptosis, redox cycling, altered calcium signaling, cytoskeletal alterations, neuroinflammation, axonopathies, myelin toxicity, delayed neuropathy, and enzyme inhibition (Masjosthusmann et al., 2018). To enable a thorough assessment of chemicals’ ANT potential, it is necessary to compile the ANT-IVB v1.0 that includes the entirety of the identified MoA, a challenge that will be addressed in our project.

To date, several assays have been established and published that cover critical KEs for neurotoxicity (Schmidt et al., 2017) but they still need refinement to meet certain criteria for regulatory acceptance of alternative methods including assessment of the domain of applicability, assay robustness and relevance, and demonstrated predictivity for adult neurotoxicity (Bal-Price et al., 2018). For the assessment of direct activation of ion channels and receptors and altered function of channels and receptors of (nociceptive) sensory neurons, several NAMs used in the DNT-IVB v2.0 can be repurposed for acute testing in mature 2D or 3D culture systems or in early life-stage zebrafish post neurogenesis. Specifically, DNT-IVB v2.0 test methods, including the myelin and BBB NAMs, cell painting in human BrainSpheres (Hartmann et al., 2023), and zebrafish learning and memory, motor system toxicity, and anxiety-like NAMs will be performed in mature cellular cultures or zebrafish embryos at time points which occur after primary neurogenesis and differentiation has occurred (R. Schmidt et al., 2013).

In addition to repurposed DNT-IVB v2.0 NAMs, several novel NAMs are being developed and applied to the ANT-IVB v1.0. One is a recently developed NAM based on hiPSC-derived nociceptor-enriched, mature sensory neurons (Holzer et al., 2022). Using this NAM after 23 days of differentiation, acute exposure to ANT reference chemicals will be carried out to evaluate the biological applicability domain of the assay. Another is the human multi-neurotransmitter assay (hMNR), which is based on hiPSC-derived mixed neuron-glia 3D BrainSpheres. The hMNR NAM assesses neuronal subtype-specific acute neurotoxicity using micro-electrode arrays (MEA) for the recording of spontaneous electrical activity (Hartmann et al., 2023). By sorting detected signals (spikes) based on their waveform, this assay allows the distinction between glutamatergic, GABAergic, dopaminergic, serotonergic, and cholinergic responses of the mixed-neuronal co-culture, allowing for an *in vitro* MoA-based assessment of ANT (Hartmann et al., 2023). In a third NAM, Ca^2+^ signaling will be assessed at the single cell level in mature central dopaminergic neurons (LUHMES cells), which extends the coverage of the KEs to another cell type and a set of functional receptors (e.g., P2X3 receptors (Apicella and Fabbretti, 2012)). A fourth NAM under development, the Peripheral Myelin Toxicity Assay (PeriMyelinTox), assesses myelin toxicity impacting peripheral sensory and motor function, and therefore addresses the key battery gap “myelination” for peripheral ANT in human cells. In this NAM, sensory or motor neurons, along with Schwann cells, will be differentiated from hiPSCs and cultivated in co-culture (Muller et al., 2018; Schenke et al., 2020; Louit et al., 2023). A novel 3D sphere format will be developed and compared to a conventional 2D format. Myelin formation will be evaluated by quantifying myelin basic protein (MBP) or myelin protein zero (MPZ) against the pan-neuronal marker β3-tubulin (TUJ1) through immunofluorescence staining and RT-qPCR (Chesnut et al., 2021). The assay will be optimized for automated high-throughput quantification of myelin post-exposure to a training set of potential myelin toxicants. The added value of both assays in assessing sensory and motor neuron myelin toxicity will be further evaluated.

To clearly define the applicability domains and assay-specific limitations, this project aims to develop a set of ANT reference chemicals that are known to affect the human brain, as well as negative compounds. This approach will ensure the unified characterization of the applicability domain of each assay and coverage of important human-relevant ANT MoA.

## 7 Outcomes and future perspectives

PARC was designed to address challenges inherent in moving from animal-based test methods to (batteries of) *in vitro* and alternative NAMs to speed up and modernize hazard identification and chemical risk assessment. Project 5.2.1e aims to improve the hazard prediction paradigm via the establishment and refinement of NAMs for DNT and ANT testing and the assembling of high performing, reproducible NAMs that provide added value into DNT and ANT test batteries. Our strategy encompasses the refinement of existing assays, generation of innovative NAMs to address identified gaps, determination of the applicability domain, and increased cost-effectiveness of lengthier assays via the demonstration of early indicators of later effects. Importantly, the development of new assays and the refinement of existing ones includes a strong focus on assay and data reliability. One critical ambition is to introduce quality control measures as described in Good Cell Culture Practices 2.0 (Pamies et al., 2022) or the GIVIMP document of the OECD (OECD, 2018). The PARC 5.2.1e consortium therefore contributes to an improved readiness, sensitivity, and overall performance of DNT NAMs to promote an increased acceptance of DNT *in vitro* and alternative assays for wider regulatory use. By the end of this project, a guidance document will be delivered that will introduce a novel framework aiming to facilitate the regulatory use of data derived from the DNT-IVB v2.0. Such work will include considerations on how DNT-IVB data may be used in the context of an IATA or weight of evidence for hazard and risk characterization. This links seamlessly to other PARC work packages that provide information on physiologically-based kinetic (PBK) modelling to convert IVB concentrations to predicted *in vivo* doses, and to risk assessment specialists that need to consider how the predicted doses can be used to set safe exposure thresholds by, for example, considering modulatory factors in AOP or by considering variabilities and specific sensitivities in exposed populations (Schmeisser et al., 2023; Suciu et al., 2023).

In contrast to DNT, there is currently no comparable battery of NAMs for ANT testing. Therefore, the consortium will create a first-generation ANT-IVB v1.0, covering major MoAs involved in human brain functioning. Looking ahead, and to respond to PARC regulatory colleagues’ requests for data on specific compound classes, 5.2.1e NAMs will be used to evaluate the potential toxicity of natural toxins, bisphenols, and per- and polyfluoroalkyl substances (PFAS). Overall, this consortium aims to offer an unprecedented opportunity to fill a longstanding gap for the commonplace assessment of neurotoxicity potential of commercial chemicals via the generation of MoA-based, robust, reproducible, fast, and inexpensive consolidated DNT and ANT testing strategies. As all of this work is conducted under the guidance of colleagues from a regulatory field and potential end users, our ambition is to revolutionize the hazard and risk assessment of DNT and ANT in Europe.

## Data Availability

The original contributions presented in the study are included in the article/Supplementary material, further inquiries can be directed to the corresponding authors.
